# GTSE1 Facilitates the Malignant Phenotype of Lung Cancer Cells via Activating AKT/mTOR Signaling

**DOI:** 10.1155/2021/5589532

**Published:** 2021-05-01

**Authors:** Fan Zhang, Jingfei Meng, Hong Jiang, Xing Feng, Dongshan Wei, Wen Meng

**Affiliations:** ^1^Department of Cardiothoracic Surgery, Affiliated Hangzhou First People's Hospital, Zhejiang University School of Medicine, 310006, China; ^2^The Second Affiliated Hospital of Xi'an Jiaotong University, Xi'an 710061, China

## Abstract

The expression of G2 and S phase-expressed-1 (GTSE1) was upregulated in human cancer. However, its expression and roles in lung cancer have not been identified yet. In our study, we reported that GTSE1 expression was statistically higher in lung tissues than in the adjacent noncancerous tissues which might be a consequence of hypomethylation of the GTSE1 promoter. The upregulated expression of GTSE1 mRNA predicted the poorer survival of the lung patients. Ectopic expression of GTSE1 in lung cancer cells significantly increased while knockdown of GTSE1 decreased cell proliferation, cell migration, and cell invasion in H460 and A549 cells. Furthermore, knockdown of GTSE1 regulated the cell cycle and promoted cell apoptosis in H460 and A549 cells. Finally, we presented that GTSE1 was able to activate AKT/mTOR signaling in H460 and A549 cells. In conclusion, these results indicated that the overexpressed GTSE1 was involved in the progress of lung cancer by promoting proliferation migration and invasion and inhibiting apoptosis of lung cancer cells via activating AKT/mTOR signaling.

## 1. Introduction

Lung cancer is one of the most common and deadly cancers worldwide [1, 2]. Lung cancer is primarily initiated from a combination of factors including smoking, genetic factors, asbestos, radon gas, and air pollution [3, 4]. The current treatment methods for lung cancer include laser ablation of malignant lesion, surgery, chemotherapy, photodynamic therapy, and radiation therapy [5]. Thus, identification of new targets for lung cancer is of great importance for prognosis prediction and therapy.

G2 and S phase-expressed-1 (GTSE1) is found in chromosome 22q13.2-q13.3 and expressed during the cell cycle S and G2 phases [6, 7]. Its common molecular function is to bind to the tumor suppressor protein p53 and impede the cancer suppressor ability of p53 in cancers [8]. Some studies further reported that upregulation of GTSE1 was frequently found in several types of human cancers [9, 10]. Upregulation of GTSE1 promoted cell proliferation and cell migration and invasion in the progress of hepatocellular carcinoma [11]. In gastric cancer cells, GTSE1 expression inhibited apoptotic signaling and conferred resistance to cisplatin [12]. The overexpression of GTSE1 suppressed cisplatin sensitivity via p53 apoptotic signaling in gastric cancer [12]. It has been confirmed that the expression of GTSE1was increased in lung cancer, and its high expression has a close relationship to the histological types [13]. However, the prognosis and roles of GTSE1 have not yet been investigated in the development of lung cancer.

In this study, we demonstrated that GTSE1 was upregulated and correlated with worse outcome in lung cancer. Ectopic expression of GTSE1 promoted whereas knockdown of GTSE1 inhibited the proliferation, migration, and invasion of lung cancer cells A549 and H460. Deletion of GTSE1 regulated the cell cycle and triggered apoptosis in A549 and H460 cell lines. We think that GTSE1 may exert the above biological functions by activating the AKT/mTOR signaling pathway in lung cancer.

## 2. Materials and Methods

### 2.1. Tissue Specimens

Lung cancer tissues and adjacent noncancerous tissues from patients with lung cancer were collected from Affiliated Hangzhou First People's Hospital, Zhejiang University School of Medicine. This study was approved by the ethics committee of Affiliated Hangzhou First People's Hospital, Zhejiang University School of Medicine. Written informed consent was obtained from all the participants.

### 2.2. Data Mining and Analysis

The online cancer microarray database, Oncomine (http://www.oncomine.org), and TCGA database were used to assess the GTSE1 transcription level in lung cancer compared with that in normal controls by Student's *t*-test.

### 2.3. Survival Analysis

Kaplan-Meier survival curves with a hazard ratio (HR), logrank *p* value (*p*), and 95% confidence interval (CI) were analyzed and plotted using the Kaplan-Meier plotter (http://kmplot.com) platform and Xen platform, which has integrated the gene expression data, relapse-free, and TCGA (The Cancer Genome Atlas), EGA (European Genome-phenome Archive), and overall survival information from GEO (Gene Expression Omnibus).

### 2.4. Cell Culture and Transfection

The lung cell lines (H146, H82, H460, A549, and H460) and human normal lung epithelial cell line (Beas-2b) were cultured in RPMI 1600 added with 10% fetal bovine serum (FBS) in a humidified incubator with 5% CO_2_ at 37°C.

### 2.5. Lentivirus Infection

The lentiviruses used to overexpress GTSE1 and control empty vector were from GeneChem (Shanghai, China), and lenti-shRNA for GTES1 knockdown and scramble shRNA were synthesized by GenePharma (Shanghai, China). Lentivirus productions were described previously [14].

### 2.6. Cell Proliferation Assay

Cell proliferation was assessed using the CCK8 counting kit (Promega). The cells were uniformly seeded in a 96-well plate at a density of 1 × 10^4^, and the OD value of the cells at 450 nm wavelength was measured at the same time for 5 consecutive days to evaluate cell viability.

### 2.7. Colony Formation Assay

Cell number was counted, and cells were seeded in a six-well plate with 300 cells per well. After culturing for 2 weeks, cell colonies were fixed using methanol, dyed with 5% crystal violet, and counted.

### 2.8. Wound Healing Assay

The cells were seeded in 6-well plates until 90% confluence. The confluent monolayers were scratched with a 200 *μ*l pipette tip to generate the wound. The debris and floating cells were removed by PBS washing. The cells were cultured for 24 hours and 48 hours for wound healing. The photographic images were taken at 0 hour, 24 hours, and 48 hours.

### 2.9. Transwell Invasion Assays

The invasive activity of the cells was detected using the transwell migration assay. Briefly, the cell culture medium containing 10% FBS was added into the lower chamber. After transduction, the cells were seeded onto the upper chamber of the transwell. The invasive cells at the lower chamber were fixed using methanol, dyed with 5% crystal violet, and imaged under a microscope.

### 2.10. Cell Cycle and Apoptosis Analysis

Based on the manufacturer's instruction, the cell cycle and apoptosis were analyzed by using the PI staining kit and Annexin-V-FITC/PI staining kit (Invitrogen), respectively. After staining, the cells were analyzed by using a flow cytometer (CytoFlex, Beckman Coulter, Fullerton, CA, USA).

### 2.11. Statistical Analysis

All statistical analyses were performed using SPSS 22.0 (SPSS Inc., Chicago, IL). The values are presented as means ± SD. The difference between the groups was analyzed by Student's *t*-test or one-way analysis of variance. *p* value < 0.05 was considered statistically significant.

## 3. Results

### 3.1. The Prognostic Value of GTSE1 mRNA Level in Lung Cancer

**Figure 1:**
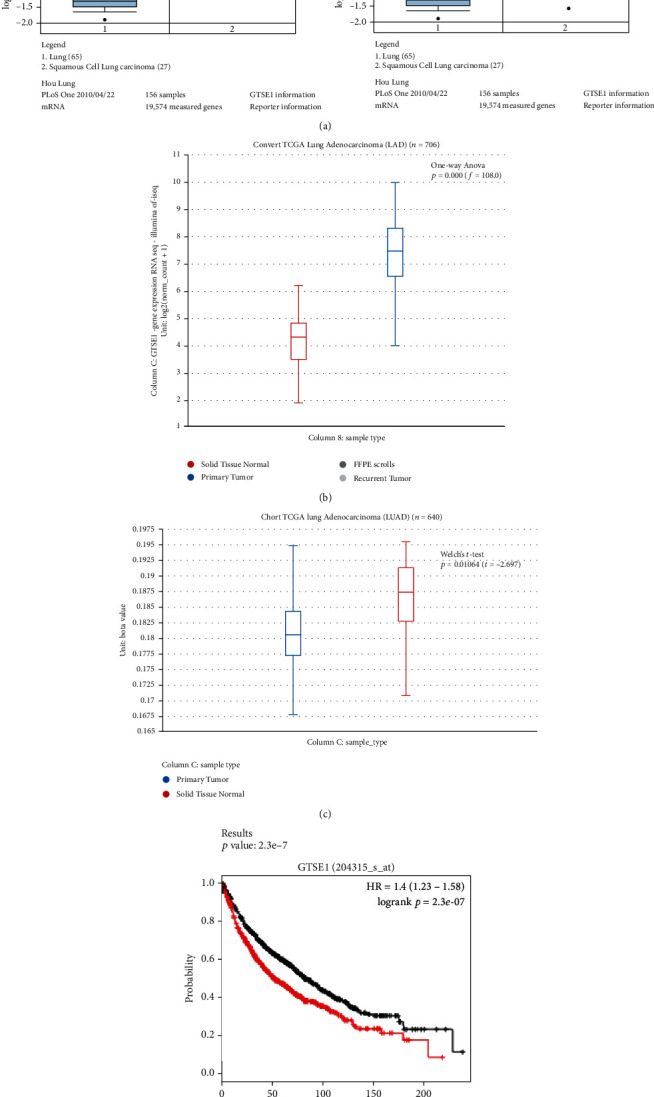
The prognostic role of GTSE1 expression in lung cancer. (a) The mRNA levels of GTSE1 in squamous cell lung carcinoma and lung adenocarcinoma were higher than those in lung normal tissues. (b) TCGA database revealed the GTSE1 expression in lung tumor samples and normal lung samples. (c) TCGA database revealed that the methylation level of the GTSE1 promoter was significantly decreased. (d) The Kaplan-Meier plotter analysis database revealed the overall survival of patients with lung cancer with high or low GTSE1 expression.

By consulting the Oncomine database, we found that the mRNA levels of GTSE1 were higher in lung cancer tissues than in normal lung tissues. The mRNA levels of GTSE1 were elevated in squamous cell lung carcinoma and lung adenocarcinoma compared with normal lung tissues (fold change: 6.204 and 3.068, respectively) (Figure 1(a)). From TCGA database, we demonstrated that the levels of GTSE1 were increased in lung cancer tissues compared with normal lung tissues. TCGA database revealed that the GTSE1 expression is also increased in lung tumor samples (Figure 1(b)). Through TCGA database, we also found that the methylation level of the GTSE1 promoter was significantly decreased (Figure 1(c)). We think that the hypomethylation of the GTSE1 promoter might be responsible for its high expression. The Kaplan-Meier plotter analysis database revealed that high GTSE1 expression predicts poorer overall survival of lung cancer patients (Figure 1(d)). Therefore, the GTSE1 mRNA expression can act as a prognostic marker for patients with lung cancer.

### 3.2. GTSE1 Is Upregulated in Lung Cancer Samples and Cell Lines

**Figure 2:**
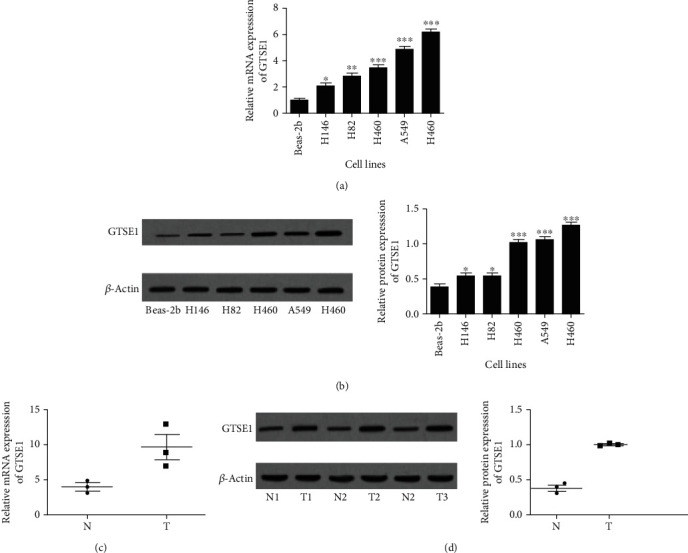
GTSE1 expression in lung cancer tissues and cells. (a) RT-PCR analysis of GTSE1 mRNA in lung cell lines. (b) Western blotting analysis of GTSE1 protein in lung cell lines. (c) RT-PCR analysis of GTSE1 mRNA levels in lung cancer tissues. (d) Western blotting analysis of GTSE1 protein in lung cancer tissues. All the values of three independent experiments were presented as means ± SD. ^∗^*p* < 0.05 and ^∗∗^*p* < 0.01.

To investigate the expression of GTSE1 in lung cancer cell lines and samples, the messenger RNA (mRNA) levels of GTSE1 in the tumor samples and corresponding normal tissues were detected by RT-PCR. The mRNA levels of GTSE1 showed significant upregulation in lung cell lines (H146, H82, H460, A549, and H460) compared with the Beas-2b cell line. In accordance with mRNA level changes, the western blotting assay revealed that the protein levels of GTSE1 were significantly increased in these cell lines (H146, H82, H460, A549, and H460) compared with the Beas-2b cell line (Figure 2(b)), especially in A549 and H460 cells. Furthermore, we detected the GTSE1 mRNA expression level in lung cancer tumor tissues. As can be seen from Figure 2(c), the mRNA level of GTSE1 was significantly increased in 3 paired cancer tissues compared with noncancerous tissues. Consistently, the western blotting assay also revealed a significant increase in the protein level of GTSE1 (Figure 2(d)). All these data demonstrated that GTSE expression was upregulated in lung cancer cell lines and tissue samples.

### 3.3. Ectopic Expression of GTSE1 Promotes Proliferation, Invasion, and Migration of Lung Cancer Cells A549 and H460

**Figure 3:**
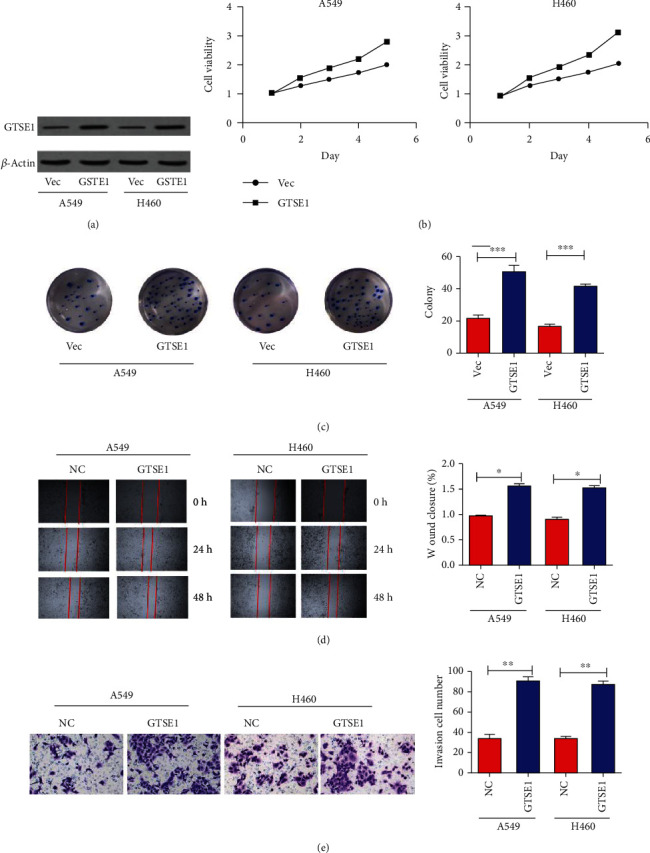
Ectopic expression of GTSE1 promotes proliferation, migration, and invasion of lung cancer cells A549 and H460. (a–e) GTSE1 was overexpressed in A549 and H460 cells. (a) The overexpression of GTSE1 in cells A549 and H460 was validated by using the WB assay. The CCK8 assay (b), colony formation (c), wound healing assay (d), and transwell invasion assay (e) were performed to evaluate cell proliferation, migration, and invasion. ^∗^*p* < 0.05 and ^∗∗^*p* < 0.01.

To explore the functions of GTSE1 in the lung cancer cell line, the lentivirus-mediated expression system was adopted to stably overexpress GTSE1 in A549 and H460 cells. We validated the overexpression of GTSE1 in the two cell lines using the WB assay (Figure 3(a)). The CCK8 assay showed that cell proliferation was promoted by GTSE1 overexpression (Figure 3(b)). The results of the colony formation assay showed that overexpression of GTSE1 increased the colony number of A549 and H460 cell lines (Figure 3(c)). The wound healing assay showed that overexpression of GTSE1 promoted the migration of A549 and H460 cell lines (Figure 3(d)). The transwell invasion assay showed that overexpressed GTSE1 significantly increased the A549 and H460 cell invasion (Figure 3(e)). These results revealed that GTSE1 could promote the proliferation, colony formation, cell migration, and invasion in lung cancer.

### 3.4. Knockdown of GTSE1 Inhibited Proliferation, Invasion, and Migration of Lung Cancer Cells A549 and H460

**Figure 4:**
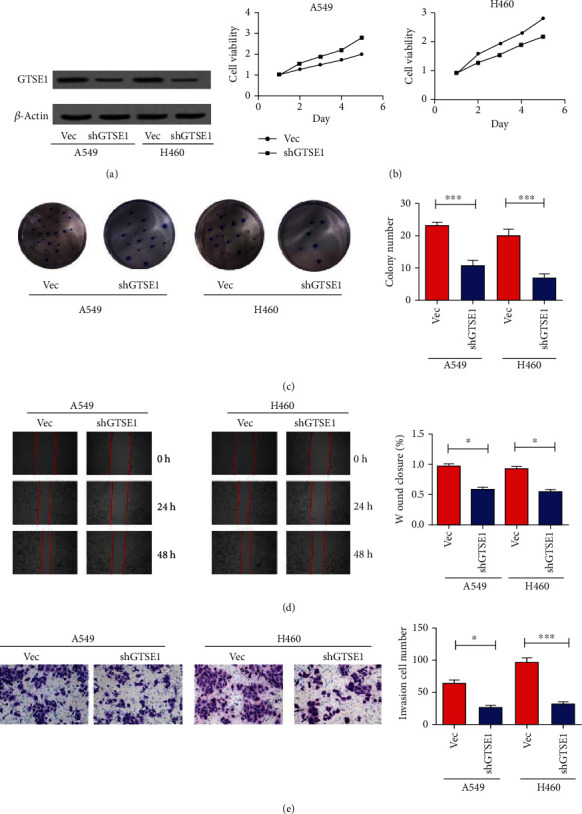
Knockdown of GTSE1 inhibited proliferation, migration, and invasion of cells A549 and H460 in lung cancer. (a–e) GTSE1 was silenced in A549 and H460 cells. (a) The silence of GTSE1 in cells A549 and H460 was validated by using the WB assay. The CCK8 assay (b), colony formation (c), wound healing assay (d), and transwell invasion assay (e) were performed to evaluate cell proliferation, migration, and invasion. ^∗^*p* < 0.05 and ^∗∗^*p* < 0.01.

To further confirm the oncogene role of GTSE1, the GTSE1 gene was silenced by shRNAs delivered by lentivirus in the H460 and A549 cell lines. We validated the silence of GTSE1 in both A549 and H460 cell lines by using the WB assay (Figure 4(a)). The results of the CCK8 assay showed that cell proliferation was inhibited by GTSE1 downregulation (Figure 4(b)). In the colony formation assay, low expression of GTSE1 inhibited the colony number of A549 and H460 cell lines (Figure 4(c)). The wound healing assay showed that low expression of GTSE1 decreased the migration ability of A549 and H460 cell lines (Figure 4(d)). The transwell invasion assay presented that silencing GTSE1 significantly inhibited the invasion of A549 and H460 cell lines (Figure 4(e)). These results demonstrated that silencing the GTSE1 expression suppressed the proliferation, colony formation, invasion, and migration of lung cancer cells.

### 3.5. Deletion of GTSE1 Regulated the Cell Cycle and Induced Cell Apoptosis in H460 and A459 Cell Lines

**Figure 5:**
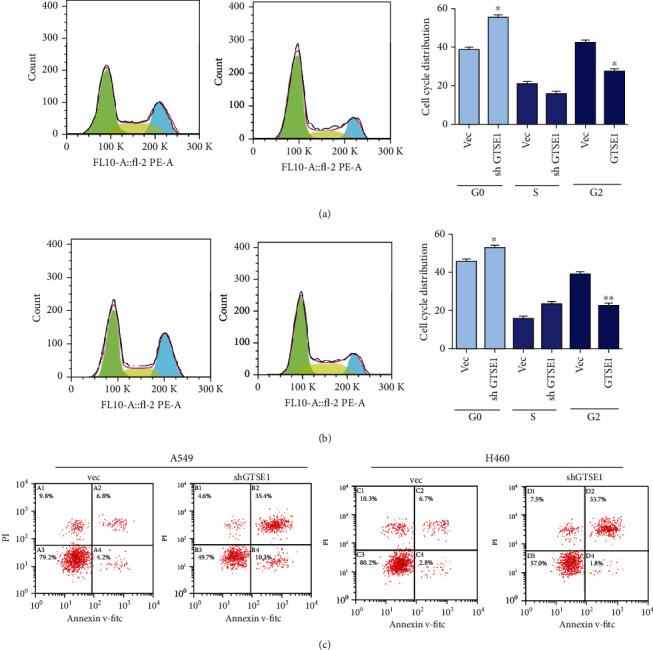
Deletion of GTSE1 inhibits the cell cycle and cell apoptosis induced in A549 and H460 cell lines. (a–c) GTSE1 was silenced in A549 and H460 cells. The cell cycle (a, b) and cell apoptosis (c) were analyzed by flow cytometry. ^∗^*p* < 0.05 and ^∗∗^*p* < 0.01.

Next, we explored the effect of GTES1 on the cell cycle and apoptosis in H460 and H460 cell lines. As shown in Figure 5, the cell cycle and cell apoptosis were measured by flow cytometric analysis. Silencing of GTSE1 inhibited the S of cell cycles and suppressed the cell cycles in H460 and A549 cells (Figures 5(a) and 5(b)). Silencing of GTSE1 induced cell apoptosis of H460 and A549 cells (Figures 5(c). These results indicated that silencing the GTSE1 expression regulated the cell cycle and induced cell apoptosis in H460 and A549 cell lines.

### 3.6. GTSE1 Suppressed p53 Expression and Activated AKT/mTOR Signaling in H460 and A459 Cell Lines

**Figure 6:**
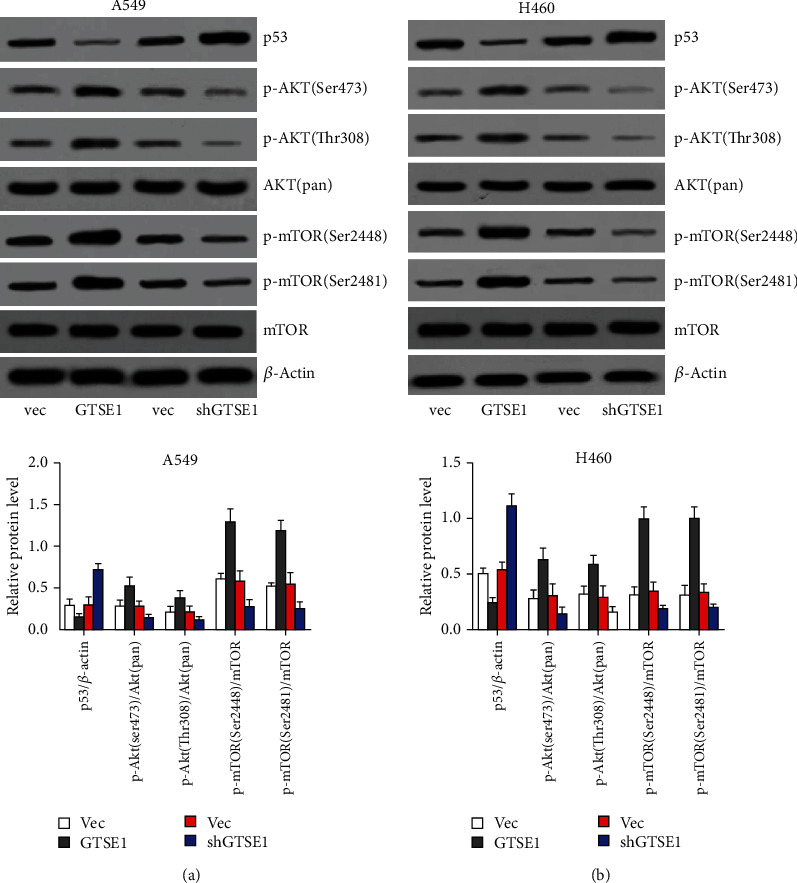
GTSE1 activated AKT signaling in H460 and A459 cell lines. (a, b) GTSE1 was overexpressed and silenced in both A549 and H460 cells. p53 protein, phosphorylation of Akt, and mTOR protein level were detected by the western blotting assay. ^∗^*p* < 0.05.

To investigate the potential molecular mechanism of GTSE1 in regulating lung cancer cells' malignant phenotype, we detected the activation of tumor-related signaling pathways. Our data showed that overexpression of GTSE1 significantly downregulated p53 protein level and promoted the phosphorylation of Akt and mTOR in H460 and A549 cell lines (Figure 6(a)). Accordingly, silence of GTSE1 significantly raised p53 protein level and suppressed the phosphorylation of Akt and mTOR in H460 and A549 cell lines (Figure 6(b)). These results indicated that GTSE1 was able to suppress p53 expression and activate AKT/mTOR signaling in lung cancer cells.

## 4. Discussion

The abnormal expressions of different genes have been explored in many cancer types and play an important role in cancer development [15–18]. In this study, we reported that the expression of GTSE1 was enhanced in lung cancer tissues and cells, and we think that the hypomethylation of the GTSE1 promoter might be responsible for its high expression. The upregulated GTSE1 was inseparable from the prognosis of lung cancer. Furthermore, the proliferation, invasion, and migration of H460 and A549 lung cancer cells were promoted by ectopic expression of GTSE1 while being inhibited by knockdown of GTSE1. Our results demonstrated that GTSE1 might function as an oncogenic trigger in the development and progression of lung cancer, which may be useful as a therapeutic target for lung cancer.

GTSE1, a microtubule-localized protein, was reported to overexpress in many kinds of human cancer and negatively regulate the functions of p53 [19, 20]. It has been reported that the high GTSE1 expression contributes to the malignant behavior of human cancer [10, 21]. In neuroendocrine tumors, overexpression of GTSE1 can augment aggressive phenotypes [22]. Consistent with these results, our results indicated that the GTSE1 expression was increased in lung cancer tissues and cell lines, and this outcome is correlated with the overall survival of patients with lung cancer [12]. In summary, GTSE1 could play a crucial role in the development and progression of lung cancer.

GTSE1 is well correlated with tumor progression [12]. GTSE1 exerts a major effect on clone formation and has direct association with promoting proliferation in hepatocellular carcinoma (HCC) cells [23]. Furthermore, our results also showed that GTSE1 knockdown inhibited cell proliferation and clone formation in A549 and H460 cells, which is in accord with the data on hepatocellular carcinoma and breast cancer. Previous studies have also suggested that GTSE1 regulates the cell cycle via promoting p53 localization to the cytoplasm. In A549 and H460 cells, GTSE1 knockdown resulted in an increased G0/G1 phase while decreased G2/M phase, which indicated that the abnormal expression of GTSE1 affected the distribution of the cell cycle.

In breast cancer, the protein levels of GTSE1 were shown to determine the migratory capacity of nontransformed breast cancer cell lines [24]. Upregulated GTSE1 correlates with tumor stage, invasive potential, and distant metastasis [25]. Consistent with this study, we reported that cell invasion and migration in lung cancer were dependent on the GTSE1 expression levels. The ectopic expression of GTSE1 promoted cellular invasion and migration, while the downregulation of GTSE1 inhibited the cellular abilities of invasion and migration.

Furthermore, we demonstrated that GTSE1 was able to suppress p53 expression and promote the phosphorylation of AKT and mTOR and then activate AKT/mTOR signaling in lung cancer cells. p53 is a well-known cancer suppressor which could inhibit the activation of many carcinogenic signaling pathways including AKT signaling [26]. So we think that GTSE1 may promote the phosphorylation of AKT by downregulating p53. AKT, also known as protein kinase B, is a known oncogene. Its abnormal overexpression or activation has been reported in many cancers and is closely related to cancer cell proliferation, survival, invasion, and migration [27, 28]. The mTOR is a Ser/Thr protein kinase downstream of AKT signaling [29–31]. mTOR is phosphorylated at Ser2448 via the Akt kinase and autophosphorylated at Ser2481 [29–31]. AKT/mTOR signaling plays a key role in promoting malignant processes of cancers [27–31]. So, we consider that GTSE1 may also facilitate tumor cell proliferation, migration, and invasion and suppress apoptosis via activating AKT signaling in lung cancer.

We acknowledged that this study did not include an in vivo assay, which was a limitation of this research. We will further study the role of GTSE1 in animal models of lung cancer in the future.

In conclusion, we defined high GTSE1 expression as a worse prognostic trigger for lung cancer patients and reported that GTSE1 acts as an oncogene by activating AKT/mTOR signaling in the lung cancer. Thus, GTSE1 may serve as a therapeutic target and novel prognostic marker in lung cancer. This study, to our knowledge, is the first to show expounding cellular function of GTSE1 protein.

## Data Availability

The data used to support the findings of this study are included within the article.
