# Correction to “GTSE1 Facilitates the Malignant Phenotype of Lung Cancer Cells via Activating AKT/mTOR Signaling”

**DOI:** 10.1155/ancp/9862178

**Published:** 2026-10-21

F. Zhang, J. Meng, H. Jiang, X. Feng, D. Wei, and W. Meng, “GTSE1 Facilitates the Malignant Phenotype of Lung Cancer Cells via Activating AKT/mTOR Signaling,” *Analytical Cellular Pathology*, 2021, 5589532, https://doi.org/10.1155/2021/5589532.

In the article, concerns were reported on PubPeer [1] regarding Figures 3 and 4. Specifically,

• The A549 graph in Figure 3b appears to be the same as the A549 graph in Figure 4b. On assessment, this error appears to have been introduced during the production process.
• The A549 and H460 images in Figure 4d seem to be identical. The authors have clarified that this was due to errors in figure preparation.

The Chief Editor has assessed the author’s response and raw data and confirmed that the results and conclusions are unaffected. The correct Figure 4 is as follows:

**Figure 4:**
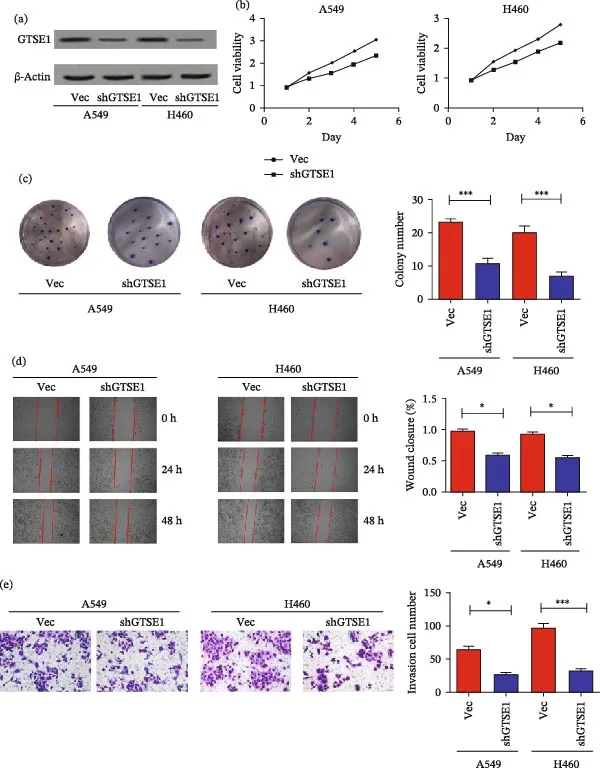
Knockdown of GTSE1 inhibited proliferation, migration, and invasion of cells A549 and H460 in lung cancer. (a–e) GTSE1 was silenced in A549 and H460 cells. (a) The silence of GTSE1 in cells A549 and H460 was validated by using the WB assay. The CCK8 assay (b), colony formation (c), wound healing assay (d), and transwell invasion assay (e) were performed to evaluate cell proliferation, migration, and invasion.  ^∗^ *p* < 0.05 and  ^∗∗^ *p* < 0.01.

We apologize for these errors.
